# Multifaceted imaging strategies for clinical trials of knee osteoarthritis—a tightly interlinked value and precision chain

**DOI:** 10.1007/s00256-025-04919-0

**Published:** 2025-04-01

**Authors:** Felix Eckstein, Ali Mobasheri, Mikael P. Boesen

**Affiliations:** 1 Center for Anatomy and Cell Biology & Ludwig Boltzmann Institute for Arthritis and Rehabilitation (LBIAR), Research Program of Musculoskeletal Imaging, Paracelsus Medical University, Strubergasse 19, 5020 Salzburg, Austria; 2 Chondrometrics GmbH, Freilassing, Germany; 3 Research Unit of Health Sciences and Technology, Faculty of Medicine, University of Oulu, Oulu, Finland; 4 Department of Regenerative Medicine, State Research Institute Centre for Innovative Medicine, Vilnius, Lithuania; 5 Department of Joint Surgery, First Affiliated Hospital of Sun Yat-Sen University, Guangzhou, China; 6 Department of Radiology, Copenhagen University Hospital Bispebjerg and Frederiksberg, Copenhagen, Denmark; 7 Department of Clinical Medicine, Copenhagen University, Copenhagen, Denmark

**Keywords:** Multifaceted, Magnetic resonance imaging, Osteoarthritis, Clinical trial, Mechanism of action, Image acquisition guide, Image analysis, Endpoint

## Abstract

Osteoarthritis is a debilitating, whole-organ disease that involves all osteoarticular tissues. No effective treatments have yet been approved by the regulatory agencies for modifying the natural history of this disease and its structural progression. In this whitepaper, we will summarize concepts of “multi-faceted” (multi-contrast) magnetic resonance imaging (MRI), with a focus on its application in osteoarthritis clinical trials. The process described here encompasses a tightly interlinked value and precision chain, where all elements must be aligned seamlessly for the trial to succeed. The procedure should begin with careful patient characterization and selection, potentially aided by (multifaceted) imaging, so that the disease (sub-) types in these potential participant will match the mechanism of action (MOA) of the medical investigational product (i.e., the drug studied). This should be followed by a comprehensive yet efficient MRI acquisition protocol, employing sequences with various contrasts that permit the characterization of imaging-based joint pathologies and evaluation of the drug’s MOA. Suitable image analysis tools must be employed, ideally adhering to regulatory compliance standards. Multiple validated endpoints can be derived from the various (multifaceted) contrasts, to be potentially combined to multi-component or composite endpoints, based on stringent a-priori rules. In conclusion, a robust value and precision chain needs to be fully functional for a disease-modifying osteoarthritis trial to be successful. Participant selection must be mindful of the drug’s MOA, and a well-aligned and custom-tailored “multifaceted” image acquisition protocol, suitable image analysis tools, and meaningful endpoints should be in place, which should match the specific image contrasts.

## Introduction

Osteoarthritis is the most common form of arthritis and affects more than 500 million people worldwide; it causes medical expenditures in the order of 2.5% of the gross domestic product [1]. It is a serious and highly debilitating whole-organ, multi-tissue disease [2], and thus far no disease-modifying osteoarthritis drug (DMOAD) has been approved. Clinical management is hence focused on controlling symptoms and function, e.g., by weight loss, strength exercise for maintaining muscle function, and pain medication. Yet, the efficacy of these measures is limited, and chronic pain management involves health risks [3, 4].

Osteoarthritis has been identified as a heterogeneous disease, i.e., in terms of severity, natural history, and treatment response [5], and may be stratified into multiple subtypes in a primary healthcare setting, i.e., into clinically apparent phenotypes [6–8]. Its heterogeneity may reflect different underlying mechanisms, i.e., molecular endotypes (molecular, cellular, immunological, genetic, and genomic features) that may drive disease pathogenesis, incidence, and progression [6–8]. (Table 1). Genotypes can influence anatomical shapes, configurations, and their pathology (i.e., morphotypes) as well as associated disease mechanisms (endotypes), knowledge of which offers opportunity to match these underlying disease mechanisms to an investigational medicinal product’s mode of action (MOA), and certain “theratypes” (therapeutic subtypes) [6–8]. (Table 1). “Imaging phenotypes” represent observable characteristics or traits (faces or “facets”) of tissues, organs, or entire organisms captured through current medical imaging techniques, and similarly to “clinical phenotypes,” these may be used for subtyping disease [9–12] (Table 1).

**Table 1:** Definitions of the key terminologies discussed in this article highlighting how multi-faceted medical imaging can play a role in analyzing or visualizing biological and clinical characteristics

Terminology	Concise definition	Relationship with medical imaging
Genotype	The genetic makeup of an organism, consisting of all the inherited genes	Medical imaging cannot directly visualize human genotypes. But it can be used to visualize genomic features in some simple animal models, to correlate visible phenotypic changes in tissues with underlying genetic mutations, or with genetic variation
Molecular endotype	A subtype of a disease defined by a distinct functional or molecular mechanism	Imaging may differentiate endotypes by revealing distinct functional changes in organs or tissues, such as inflammation and metablic alteractions, as long as there are biochemical and biological markers that reflect the activities of the endotypes
Anatomical morphotype	A classification based on the physical or anatomical characteristics of an organism or part of the anatomy	In musculoskeletal research, high-resolution imaging can visualize and compare morphotypes by examining anatomical structures, features, and variations, such as different bone shapes and micro-structural features
Clinical phenotype	The observable traits of an organism, resulting from the interaction between its genotype and environment	In a primary healthcare setting, medical imaging plays an indirect role in confirming the existence of disease. Radiography, MRI and micro-CT can provide further information about structural damage to joint tissues, but do not illuminate clinical phenotypes. By definition, clinical phenotypes do not require advanced imaging
Imaging phenotype	Observable characteristics or traits of tissues, organs, or entire organisms captured through medical imaging techniques	Medical imaging is of course central to defining, imaging “phenotypes, as it reveals structural, functional, immuno-metabolic, and inflammatory traits. These so-called imaging phenotypes help in disease diagnosis, monitoring, and treatment personalization. However, these must not be confused with clinical phenotypes
Theratype	A classification of patients or diseases based on their response to a specific therapy. A therapeutic subtype	Imaging is crucial for identifying and monitoring theratypes. It allows clinical researchers to select particular study participants, and to observe how tissues and organs respond to specific treatments. This can guide the development of new drugs and personalized therapies. In the field of OA research, however, this paradigm is yet to be developed

Multi-faceted imaging refers to application of a single imaging technology (or modality), such as MRI, that can exploit multiple image contrasts, from different sequences. These may obtain comprehensive views of tissues or organs, based on the design of an image acquisition protocol [13, 14] that consists of a specific choice of MRI sequences. Compared with radiography or computed tomography, MRI has greater potential for “multifaceted” imaging, by combining various imaging sequences with different image contrasts that provide a more comprehensive and deeper understanding of the structural pathology than techniques that display one dominant contrast only [9, 12, 13]. This “holistic” approach can permit more precise diagnoses and prognoses, better sensitivity in monitoring disease progression, and superior detection of responses to osteoarthritis treatment [15]. Further, maintaining consistency of spatial information when relying on only one imaging method warrants high precision and coherence of subsequent data analysis and interpretation.

**Fig. 1:**
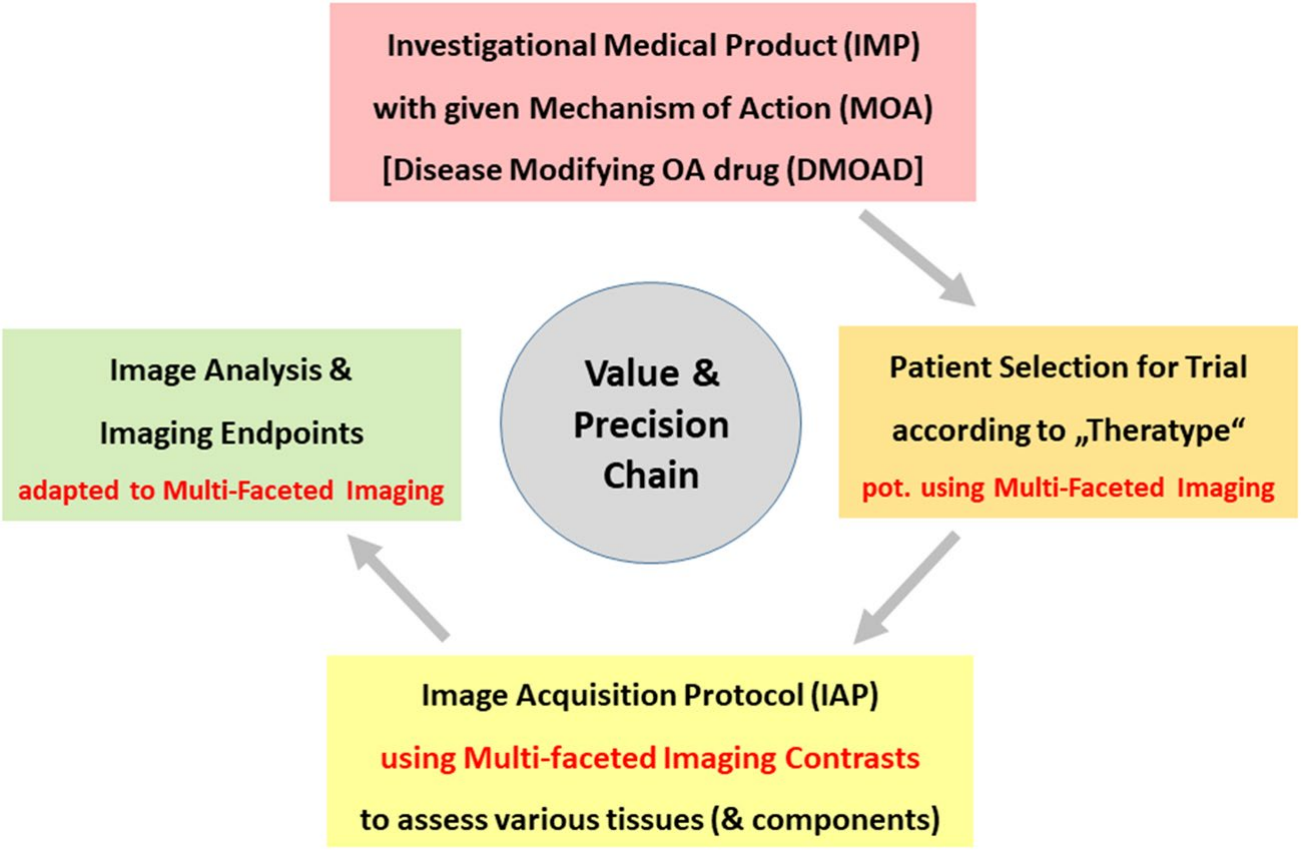
Flow chart depicting multi-faceted imaging in osteoarthritis drug (DMOAD) development

For an osteoarthritis clinical study to be successful, use of multi-faceted imaging requires a tightly interlinked value and precision chain (Fig. 1), where each element needs to match the previous and next one, respectively. Participant selection should begin with phenotyping (clinical and/or imaging) and with collecting detailed information on the disease history. In interventional trials, ideally this would be followed by omics-assisted endotyping to identify pathogenic drivers, and to ensure identification of an “endotype-theratype” combination that represents a treatable disease subtype [6–8]. This should ideally then be amendable to the drug’s MOA and warrant structural improvement. The corresponding phenotype must be appropriately depicted by the specific image acquisition protocol [13, 14], and the analytic tools for image assessment must match the image contrast, resolution, and orientation [9, 10, 15, 16]. Eventually, with “multifaceted” imaging permitting to attain more than one (imaging) endpoint, it becomes possible to combine multiple assessments into multi-component or composite endpoints [17–19] (Fig. 1).

## Objective

The goal of this white paper is to discuss outline concepts and mechanisms of “multifaceted” knee MRI in the context of DMOAD clinical trials. We here describe a tightly interlinked value and precision chain that ranges from selecting patients mindful of the drug’s MOA [6–8], designing a patient- and MOA-orientated MRI acquisition protocol [13], and aligning the image analysis tools and choice of endpoint(s) with that “multifaceted” imaging approach [9, 15] (Fig. 1). This white paper is directed at radiologists and non-radiologists, physicians, and non-physician health care providers, with an interest in clinical trials in osteoarthritis, with or without an intervention. Although the paper dominantly refers to pharmacological (DMOAD) treatment, its principles also apply in many aspects to other therapies, such as lifestyle/rehabilitative, surgical, or other interventions.

## Concepts and mechanisms

### MRI contrasts

#### Weighting

**Fig. 2:**
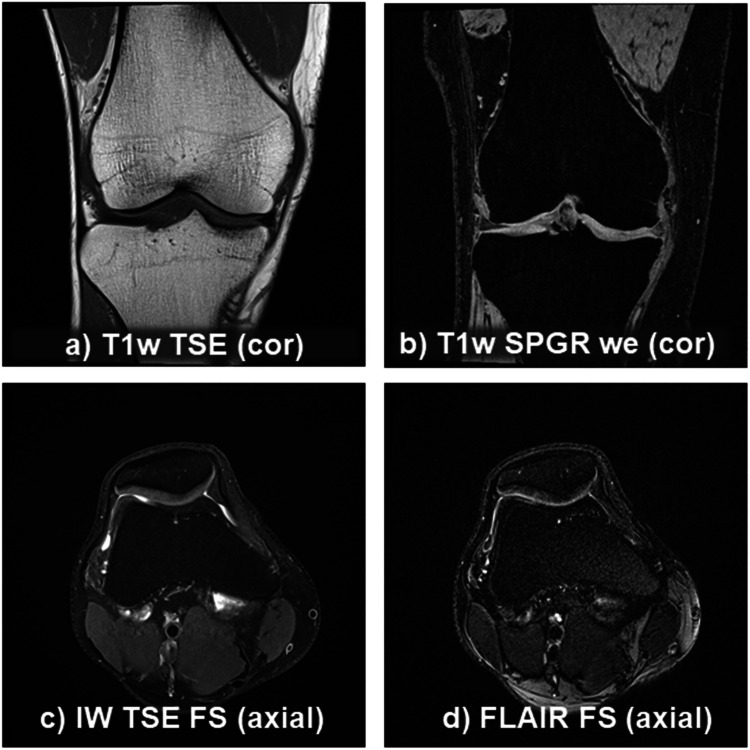
Multifaceted MRI with different contrasts commonly used in osteoarthritis imaging, visualizing **a** the general anatomy, collateral ligaments, bone pathology, etc.; **b** cartilage morphometry (thickness, volume, surface, and denuded areas, etc.); **c** effusion synovitis, femora-patellar cartilage pathology, bone marrow lesions (BMLs), etc.; and **d** separation of thickened synovium and effusion

**Fig. 3:**
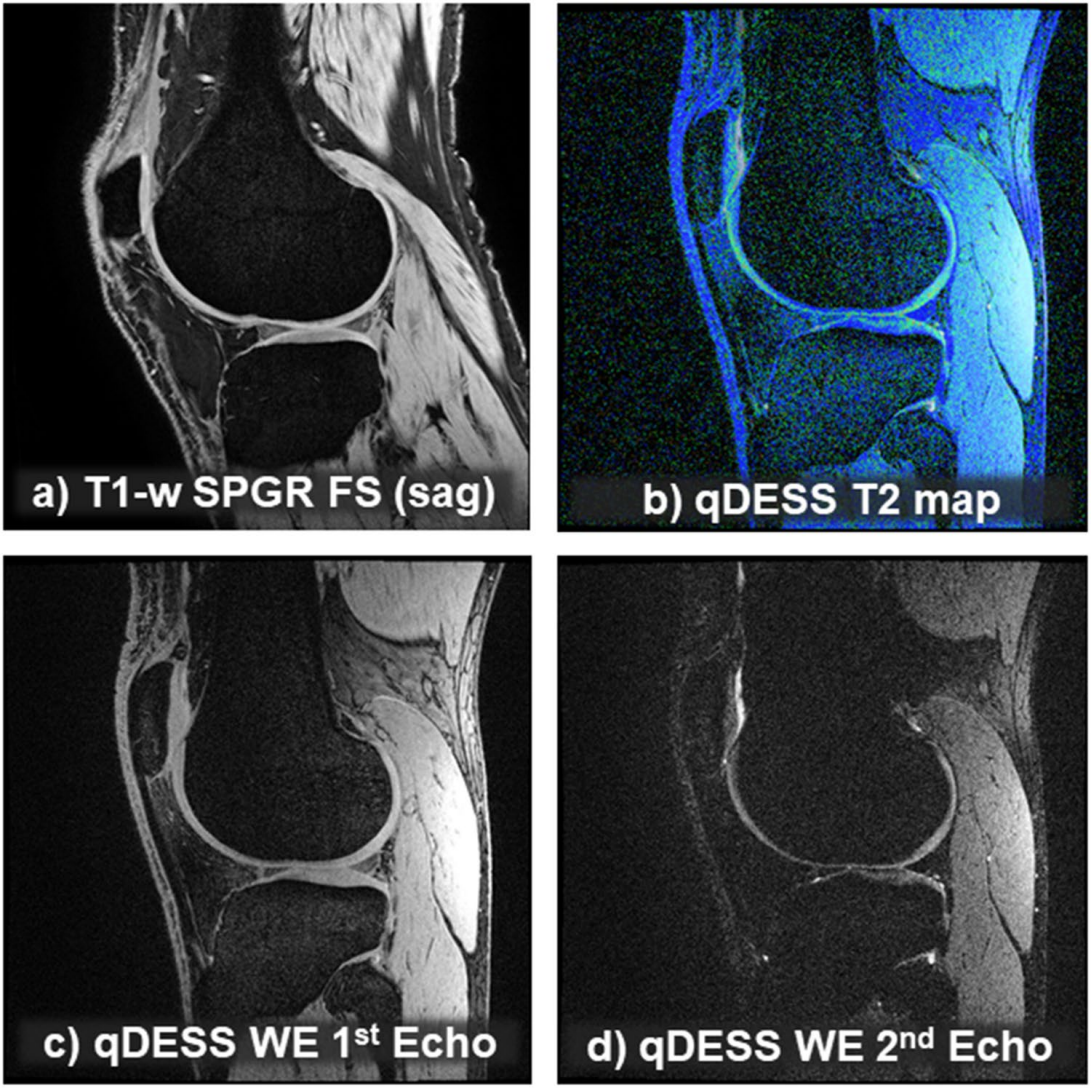
Multifaceted MRI to simultaneously obtain quantitative cartilage morphology and relaxometry (transverse relaxation time [T2]), also suitable for bone size and shape analysis, for meniscus morphometry (if reconstructed in the coronal plane) and potentially for analysis of Hoffa synovitis (under investigation). **a** Sagittal T1-weighted spoiled gradient echo sequence with fat suppression. **b** T2 map derived from quantitative dual echo at steady state sequence with water excitation (qDESS) with water excitation (WE), as depicted in **b** and **c** (two echos). **c** qDESS WE (1st echo). **d** qDESS WE (2nd echo)

The options to create different contrasts with MRI are almost infinite and beyond the scope of this paper; therefore, we will focus on those that are most commonly used in osteoarthritis imaging (Figs. 2 and 3) [9, 13]:

- T1-weighted (T1w) turbo spin echo (TSE) emphasizes differences between the T1 (the longitudinal relaxation time) of tissues as contrast mechanism. It relies on a short repetition time (TR, 300–700 ms) and echo time (TE, 10–20 ms), so that adipose tissue appears hyperintense (bright) and fluid/edema/effusion shows up hypointense (dark).
- T2-w TSE, in contrast, emphasizes differences in the T2 by using a long repetition (TR > 2000 ms) and echo time (TE, 60–210 ms), with adipose tissue and fluid appearing hyperintense. To distinguish fluid and adipose tissue, T2-w TSE commonly applies spectral fat saturation (see below) to null the fat signal. Thus adipose tissue appears dark whereas edema, Hoffa synovitis, and effusion synovitis appear hyperintense [16].
- Often though, evaluation of articular tissue pathology requires a “neutral” weighting, with proton density (PD)-w emphasizing the concentration of hydrogen protons as tissue contrast. PD-w uses a long repetition (TR, > 2000 ms) and short echo time (TE, 10–20 ms), minimizing both T1 and T2 effects [20].
- Lately, intermediate weighted (IW) TSE has superseded T2-w and true PD-w sequences in musculoskeletal imaging [3, 5]. IW-w relies on intermediate repetition (TR, 1500–2500 ms) and echo times (TE, 40–60 ms), to balance out T1 and to include some T2 effects [20]. It provides superior tissue differentiation than T2-w or true PD-w, although distinction in the literature is fuzzy, and IW is often termed PD-w imaging [13]. With IW contrast, fluid-based tissues such as synovitis, effusion, Hoffa synovitis [16], bone marrow edema [21], and cysts are depicted relatively hyperintense, albeit not as bright as with T2-w imaging; adipose tissue shows up hyperintense if no fat-suppression is applied [20] (see below).

#### Fat- or water-suppression

Suppression of signal from either the adipose tissue or the fluid (Figs. 2 and 3) is a widely exploited contrast mechanism in musculoskeletal imaging [22]. Fat suppression may be technically achieved with different techniques: i.e., the chemical shift-based methods, e.g., chemical shift selective (CHESS) fat saturation (FS), selective water excitation (WE), DIXON (no acronym, named after Dr. W.T. Dixon), or—on the other hand—the inversion recovery (IR) techniques (e.g., short tau inversion recovery [STIR]) [22]. Recently, hybrid sequences have been developed that combine chemical shift selective and inversion recovery for fat-suppression, such as spectral pre-saturation with inversion recovery (SPIR), or spectral attenuated inversion recovery (SPAIR).

- The chemical shift selective FS method can be used with various MRI sequences and applies a frequency-selective pre-pulse to only excite the fat-bound protons, followed by a gradient that dephases (nullifies) their signal and thus increases the signal from water protons [22, 23] (Fig. 2). Fat suppression adds only little additional acquisition time, but the method requires magnets with a field strength of ≥ 0.6 Tesla and is sensitive to magnetic field inhomogeneity, potentially causing incomplete fat-suppression in some regions (e.g., the peripheral skeleton). Techniques such as short tau inversion recovery may be preferable since they are more robust to magnetic field inhomogeneity due to using a 180° inversion pulse. They can also be employed with low field MRI (< 0.5 T), although this renders the acquisition time substantially longer. The newer hybrid techniques (spectral pre-saturation with inversion recovery, or spectral attenuated inversion recovery) therefore are more frequently applied for effective fat suppression in the clinical settings and in clinical trials. Yet, as is the case for short tau inversion recovery and DIXON, they have so far not been used in DMOAD intervention trials.
- Fat suppression with gradient echo imaging has been widely used for quantitative cartilage imaging [15, 24]. A 3D T1-w high-resolution gradient echo sequence has been combined with fat suppression, rendering adipose tissue hypointense (including bone marrow), despite T1-weighting, and the cartilage relatively hyperintense [23]. This approach clearly delineates the bone-cartilage-interface by eliminating artifacts that occur from interaction between adipose-tissue (bone marrow), subchondral bone plate (no free protons), and water-bound protons (cartilage matrix) (Fig. 2). Because fat and water protons display slightly different resonance frequencies (chemical shift), their signals can be in or out-of-phase (depending on TE) and may cancel each other out. This results in loss of signal (signal void) at the bone-cartilage-interface, showing up as a more or less broad “dark zone” that precludes accurate identification of the bone cartilage interface (Fig. 2). 3D T1-w fat-suppressed gradient echo is a way to overcome this limitation [23], and has been technically validated for cartilage morphometry vs. several reference methods [15]. It shows cartilage hyper-intense, with good contrast versus subchondral bone, the joint cavity, the meniscus, and other adjacent tissues, but is not as sensitive to cartilage lesions as the IW TSE fat suppression sequences [9, 23].
- Direct water excitation is more efficient than fat suppression; it selectively excites the water molecules but not the adipose tissue protons, since these resonate with a slightly different frequency (Fig. 3). This 3D technique comes with shorter acquisition time and is less sensitive to magnetic field homogeneities, and has shown high agreement of quantitative cartilage measurements with previous validation studies [25, 26]; it is now the most common technique applied with 3D T1-w gradient echo, particularly in the context of quantitative cartilage and bone imaging [15].
- Fluid-attenuated inversion recovery (FLAIR) [13, 27–29] is a 2D technique for fluid signal suppression, often employed in neuroimaging. It suppresses signal from the cerebrospinal fluid and reveals lesions around fluid-filled spaces, such as the ventricles. It uses an inversion pulse followed by a delay, the inversion time, which is chosen corresponding to where the fluid signal passes through “zero” (the “null point”; no signal). FLAIR has been proposed as a non-contrast alternative to contrast enhanced (CE) imaging for visualizing the inflamed synovium (synovial thickening) and fluid (effusion) in knee osteoarthritis [13, 27–29]. Whereas with IW-w TSE, both structures appear hyperintense and indistinguishable, FLAIR can assess both separately (Fig. 2, and see below).

#### Cartilage transverse relaxation time (T2)

Apart from MRI sequences used for tissue “morphometry,” there also has been overwhelming interest in using those for relaxometry, i.e., “in vivo histology.” Cartilage T2 (the transverse relaxation time) for instance, was shown to reflect matrix hydration and collagen [30]. T2 maps can be generated by various TSE or gradient echo techniques [31], the most widely used method being multiple echo spin echo (MESE). The latter commonly uses 4–8 TEs to derive a T2 value on a voxel-by-voxel basis. Recently, a more versatile 3D gradient echo-based sequence, quantitative double echo at steady state (qDESS), has been introduced for this purpose, combined with water excitation (Fig. 3) [13, 15, 32–34]. qDESS is a research sequence and not commonly available on clinical MRI scanners, but it can be installed on most MRI magnets by application specialists after obtaining research agreements with the respective vendor. qDESS generates two echoes per repetition time, separated by a spoiler gradient, permitting one to compute T2. The first acquisition displays mixed T1/T2 contrast similar to PD images, whereas the second exhibits T2-w/diffusion-weighted contrast (Fig. 3). Conventional DESS [14, 15, 35] generates a single (fused) image with mixed T1-/T2-contrast, enabling morphometry but not relaxometry (T2). Yet, with both images stored separately (qDESS), a voxel-by-voxel fit can generate the desired high-resolution T2 map. This approach has been validated versus single echo spin echo T2, the gold standard for this type of measurement [13, 15, 32–34].

#### Contrast enhancement

**Fig. 4:**
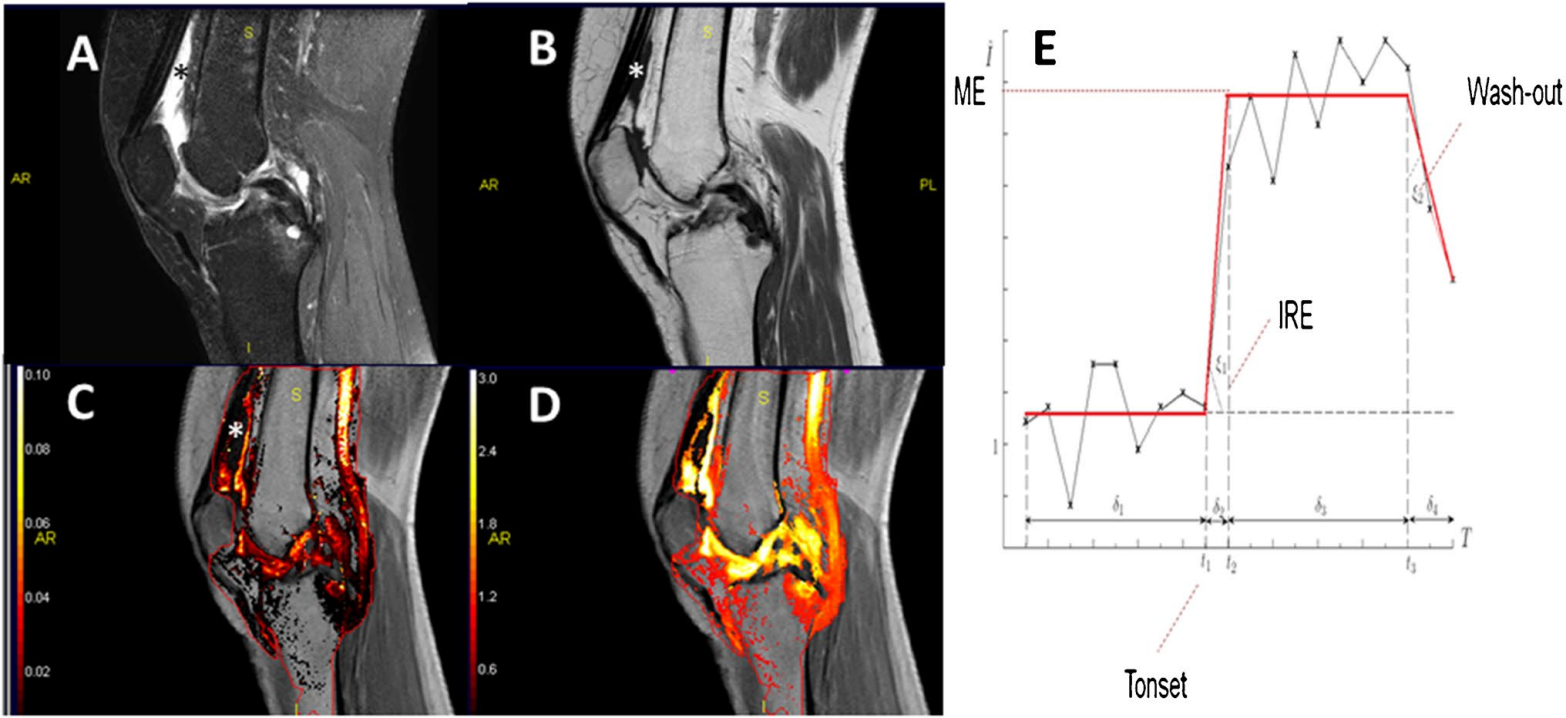
Contrast enhanced multifaceted MRI: Mid sagittal image in a patient with moderate knee osteoarthritis (Kellgren Lawrence grade 3) with moderate supra-patellar effusion-synovitis in the suprapatellar area, Hoffa synovitis, and a cyst in the posterior part of the tibia (surrounding bone marrow edema. **A** IW fat suppression sequence. **B** T1-w non-fat saturated contrast enhanced (CE). **C** T1-w dynamic contrast enhanced (DCE) gradient echo (GE) MRI analyzed using Dynamika® (Image Analysis Group,London UK) with superimposed initial rate of enhancement (IRE) map, highlighting the rate of contrast uptake in % intensity increase, until maximum enhancement (ME) is reached. **D** Same T1-w DCE-MRI GE MRI with ME map superimposed. **E** Graph showing how the time intensity curve (TIC) is calculated for each voxel using Dynamika®, the *x-*axis represents the time, the *y-*axis the signal intensity. T-onset is the time of CE. Color bars in **C** and **D** reflect low initial rate of enhancement or ME as dark red, and high values as bright yellow. Enhancing synovium has a heterogeneous location specific CE and perfusion/inflammation pattern most notable in the initial rate of enhancement map (**C**). Effusion and synovitis marked with* in **A**–**C** are indistinguishable in **A** but can be separated in **B**–**D** since effusion does not take up contrast

In most osteoarthritis and DMOAD studies, MRI is performed without the use of intravenous gadolinium (Gd) contrast enhancement (CE) for operational reasons [13]. However, synovitis and effusion are ideally depicted by (2D or 3D) T1-w fat suppressed CE-MRI [16], with several dedicated scoring and measurement systems being available [13, 16]. Dynamic CE (DCE) MRI may be applied while injecting gadolinium, for advanced assessment of synovitis and inflammatory processes in the joint [36], including the infra-patellar fat pad (or “Hoffa”) [37] and the bone marrow [38, 39]. The technique is based on rapid serial acquisitions of either 2D TSE or preferably T1-w GE images of the same anatomical area. Ideally, it should be applied at a temporal resolution of < 10 s, before, during, and 4–5 min after intravenous injection of gadolinium. When gadolinium reaches the joint, the MRI signal intensity increases linearly with change in gadolinium tissue concentration. DCE-MRI can be further analyzed by pharmacokinetic methods, such as a two-compartment model: the “extended Toft method” permits one to calculate the volume transfer constant (Ktrans), reflecting the rate of gadolinium contrast effusion from the blood plasma into the extravascular extracellular space of the tissue. Alternatively, a Heuretic semi-quantitative “signal versus time intensity curve” can be deployed [36]. Various curve profiles are used to calculate the rate of contrast enhancement, the maximum enhancement, the time to peek, the area under the enhancement curve, and the potential washout rate. To study specific tissues, the gadolinium contrast uptake and perfusion characteristics in each voxel of the DCE-MRI dataset require outlining the target tissue within a region of interest (ROI), throughout which the enhancing tissue (e.g., synovium) is present [36]. Intensity curves that display a rapid and extensive “intensity increase” (rate of enhancement), “plateau” (equilibrium), and “washout” during the first 5 min are typical for tissues with high perfusion and leaking vessels, seen in active inflammatory tissue and tumors. The above variables are specific and useful for quantifying perfusion in the synovium, the Hoffa, or the bone marrow on a continuous scale, and have been shown to display stronger correlations with pain [40] and histological markers of inflammation [41] than conventional methods of static or even CE MRI [16] (Fig. 4). Further, they appear more sensitive in detecting the early inflammatory treatment response than static or CE MRI [42].

#### Proteoglycan-specific contrast of articular cartilage

Another important set of contrast mechanisms in osteoarthritis imaging is that for assessing proteoglycans (or their glycosaminoglycans [GAGs] subcomponent), both being strongly negatively charged [43–45]. These encompass the following:

- Delayed Gadolinium-Enhanced Magnetic Resonance Imaging of Cartilage (dGEMRIC) [43–45], measuring the “delayed” uptake of negatively charged gadolinium about 90 min after intravenous injection and active movement of the patient, inversely related to the content of the negatively charged glycosaminoglycans. However, due to the complexity of the (delayed) acquisition and gadolinium injection, and the long imaging time, this setup has not been commonly used in DMOAD trials.
- Other techniques for glycosaminoglycan quantification without the use of i.v. gadolinium encompass T1-relaxation time in the rotating frame (T1rho) [31], depicting interactions between water- and macro-molecules (e.g., glycosaminoglycans) in the extracellular matrix. Glycosaminoglycan chemical exchange saturation transfer (gagCEST) [46] uses chemical exchange between glycosaminoglycans and water protons to enhance the imaging signal, whereas sodium MRI (Na-23 MRI) [47] directly measures the content of the positively charged sodium ion in the matrix rather than hydrogen, known to positively correlate with the concentration of negatively charged glycosaminoglycans. Common to all methods is that they have not been used in DMOAD trials, as they require high field scanners, dedicated hardware (coils), and sequences that are not readily available outside a few academic centers.

### Patient selection strategy

In osteoarthritis clinical trials, patient selection is commonly done based on clinical data (symptoms above a certain level) [48] or using radiography, determining the structural status of the joint (e.g., Kellgren Lawrence Grade [KLG] [49], atlas-based joint space narrowing grades [JSN] [50, 51], or [medial] joint space width [JSW] [48]. Other features by which a study population may be selected/enriched using MRI are the presence of synovitis by dynamic/static CE-MRI [36], potentially FLAIR [13, 27–29], or ultrasound [16], which is important when studying a drug with an anti-inflammatory MOA [52]. An elaborate MRI-based patient-selection tool is ROAMES (Rapid OsteoArthritis MRI Eligibility Score) [11]. It has been “validated” in Osteoarthritis Initiative data using sagittal IW 2D TSE with fat suppression, coronal IW 2D TSE, and sagittal 3D DESS with water excitation, including coronal and axial reformats. The system thus uses three different contrasts for (multifaceted imaging) classification. The reading instrument strives at defining structural participant eligibility by focusing on cartilage, meniscus, synovium (inflammation), and bone (osteophytes). It also delivers diagnoses for exclusion, such as meniscal root tears, osteonecrosis, subchondral insufficiency fracture, tumors, malignant marrow infiltration, and acute traumatic changes [11]. The selection process may be extended, for instance, by excluding higher grades of cartilage lesions, assuming that more severe pathology may respond less well to therapy as does mild or moderate structural disease, or by eliminating knees without cartilage pathology, presuming that they will not display sufficient structural change over time to be able to demonstrate a treatment effect vs. placebo. To include participants with synovitis and study how a DMOAD may affect inflammation, the use of CE-MRI (ideally DCE-MRI) or FLAIR may be considered [36]. Generally, it is worth noting that screen failure rates are already substantial when using radiographic inclusion criteria, such as JSN [48, 50, 51]. Whereas its precise impact on screen failure rates is unknown, application of additional MRI selection criteria may imply greater recruitment failure, higher trial cost, longer MRI waiting-lists, and the specific regulatory label obtained by the drug to be narrowed. Whether more advanced inclusion methodology than that currently available based on radiography will eventually enhance the chances of selecting patients tailored to a specific MOA, and of seeing a stronger structural and/or symptomatic response to therapeutic intervention remains to be established.

### Multifaceted image acquisition protocols for clinical trials

Most important in (multifaceted) imaging is a well-designed image acquisition protocol, i.e., a series of sequential acquisitions within one scanning session. Whereas analytic assessment tools for scoring and measurement, and the relevant endpoints can be adapted after the actual acquisition has been completed, failure in the acquisition protocol is disastrous and cannot be corrected later, since relevant observations can only be made on imaging data obtained during the trial period. Analytic methods and endpoints should preferably also not be changed in a pivotal trial for statistical and regulatory reasons, but in case of failure can still be “rescued” for an exploratory analysis.

An image acquisition protocol must balance the number of MRI sequences and contrasts used, enabling all specific image assessments that are tailored to specific study questions, and enabling the assessment of a treatment effect of the drug of interest, within the limited time that the patient is able or willing to tolerate [13, 14]. The image acquisition protocol of the Osteoarthritis Initiative has been well established [14], encompassing 2D IW TSE with and without fat suppression for semi-quantitatively scoring structural pathology, and 3D high-resolution spoiled gradient echo and DESS for quantification of cartilage morphology [35]. The DESS also facilitates meniscus (extrusion) morphometry [53–55] and measurement of bone size and shape [56, 57]. A multi-echo-spin-echo (MESE) sequence was also acquired to measure T2 relaxation, related to cartilage matrix composition; yet, the Osteoarthritis Initiative image acquisition protocol took 58 min (both knees), exclusive of patient and machine set-up [14]. Note the Osteoarthritis Initiative was an “academic” and exploratory study [14] and not targeted at setting up a fast and efficient acquisition protocol for an interventional clinical trial.

For this reason, a 30-min net acquisition time, state-of-the-art protocol, has been published more recently, applicable in both early or advanced disease [13]. The protocol has been developed for the PROTO (advanced PeRsOnalized Therapies for Osteoarthritis) clinical trial, with a focus on assessing synovitis [13]. As virtue of a “multifaceted” approach, the protocol supports multiple semi-quantitative and quantitative endpoints, pertinent to a multitude of pathological processes in the (peri-)articular tissues. Although considered the gold standard for synovitis imaging, use of intravenous gadolinium contrast needs to be justified by the specific MOA, and is mainly to be used in relevant inflammatory DMOAD trials. It requires monitoring by a physician and is associated with higher cost, additional patient burden, potential health risks [13], and is prohibited in patients with reduced kidney function. Given these operational challenges, the FLAIR sequence [13, 27–29] may represent a non-contrast alternative in measuring the thickened synovium and fluid separately, after further exploration and validation. This may improve on the current scoring of “effusion-synovitis” that is based on combined (indistinguishable) assessment of the synovium and the enclosed effusion taken together [16]. The suggested 30-min acquisition protocol encompasses 2D IW TSE fat suppression acquisitions in several orientations [13] similar to the Osteoarthritis Initiative. These enable evaluation of most of the relevant structural pathologies (including Hoffa- and effusion-synovitis), commonly assessed using the MRI Osteoarthritis Knee Score (MOAKS) [9, 58]. A coronal 2D T1 TSE non-fat suppressed technique is added to evaluate medial and lateral osteophytes, collateral ligaments, loose bodies, and bone pathology (e.g., subchondral sclerosis, subchondral insufficiency fractures, bone marrow infiltration, bone infection, and bone fracture). For time optimization, a novel qDESS sequence [13, 15, 32–34] was suggested as the “powerhouse” for quantitative measurement of joint structures, enabling analysis of cartilage morphology (thickness, volume, subchondral bone areas, and denuded areas) [15], laminar (deep and superficial) cartilage T2 [32–34], meniscus morphology and position (e.g., extrusion area and distance [53–55]), bone size and bone shape [56, 57], and potentially Hoffa synovitis [59, 60]. Such use of a well-designed (multifaceted) image acquisition protocol is imperative for an interventional drug (DMOAD) trial, but also highly beneficial to the study of non-pharmacologic (lifestyle/rehabilitative, surgical) interventions, and to epidemiological studies.

### Image analyses and endpoints pertinent to multi-faceted imaging

In contrast to computed tomography, MRI accommodates various image contrasts, to which the analysis methodology must be closely adapted. The human eye has been trained in evolution over millions of years, to identify features under many different circumstances. Manual segmentation by trained readers hence is very “robust” against such variation (not only between protocols, but also within protocols, i.e., within a multicenter study that involves many sites), to ensure that the study is representative [51]. Yet, when evaluating “change over time” for cartilage morphology in knee osteoarthritis trials, a reader bias was observed, so that readers must be blinded to the order of MRI for the accurate rate of cartilage change to be determined in the study participants [61]. The magnitude of response to the treatment effect, however, was the same in both reading schemes [61]. Similar considerations may apply for other quantitative and semi-quantitative assessments, but have not yet been studied.

Lately, deep learning has emerged as a powerful technology for performing fully automated segmentation, e.g., convolutional neural network algorithms [15, 62–65]. These are robust to a potential bias for baseline or follow-up status [63, 65], but sensitive to differences in contrast, texture, and resolution between the images [62]. Segmentation models hence have limited transferability from one “contrast” to another, and must preferably be trained on images with identical (or at least similar) contrast, texture, and resolution as the designated target images [62, 63]. Again, this requires robust and consistent image acquisition protocols across studies, to reduce variation, but also flexibility in the use of these models. This comes with a relatively high workload of training and validating different models (using manually segmented reference sample data) in comparison with other imaging techniques, where contrast is more consistent (i.e., Hounsfield units in computed tomography). Unsupervised learning may overcome these issues, as learning occurs from the raw images directly without any expert annotation, but these have not yet been applied to osteoarthritis clinical studies.

Multi-faceted imaging permits application of multiple endpoints derived from the same imaging modality [17–19]. From the image acquisition protocol, various assessments may be derived that are combined to either a multi-component (all factors contributing to the overall result in a specific manner, defined a priori), or a composite endpoint (any of the factors determines the overall result alone, when exceeding a defined threshold) [66], as reviewed recently [67]. Based on several potential candidates, Harkey et al. [68], for instance, defined a “disease activity” multi-component endpoint from the sum of bone marrow lesion and effusion synovitis volumes, and a “cumulative cartilage damage metric” from a series of local cartilage thickness measures. These were found to be associated with accelerated incident radiographic knee osteoarthritis [68]. The “disease activity metric” was more strongly related to symptomatic progression, whereas the “cumulative cartilage damage metric” was more strongly related with radiographic progression [69]. These are just some examples of a sheer infinite number of potential combinations of endpoints that can be used to diagnose osteoarthritis, predict its progression, and evaluate the efficacy of interventions [10], each after careful validation.

## Discussion and conclusions

Given the high prevalence of osteoarthritis and its impact on patients and the health care system, defining a systematic and uniform approach to evaluate the response to potential DMOADs can be beneficial; yet, such a proposition must also take into account the MOD of the drug studied, in order for the trial not to fail. In this paper, we describe the potential role of “multi-faceted” MRI in the context of osteoarthritis clinical trials. Multi-faceted imaging is a unique strength of MRI, due to its incredible and almost limitless ability of creating different contrasts. This entails tremendous potential for highlighting specific tissues, components, pathologies, and therapeutic effects. Here we have limited the multi-faceted imaging focus on those MRI sequences that have been used in DMOAD trials already, or hold promise for an application in the not too distant future, while being sufficiently robust for realistic implemented in a clinical trial environment. In this context, plenty of (other) “multi-” terms exist in the field of medical imaging, each highlighting different aspects of how to apply imaging in a complex, but integrated manner.

- Multi-dimensional imaging describes the acquisition and representation of image data that extends beyond those of two- or three-dimensional views. It captures additional dimensions of data, such as time, contrast, function, or else.
- Multi-planar imaging provides views in multiple planes (i.e., axial, sagittal, coronal, oblique), to enable more comprehensive radiological visualization and evaluation.
- Multi-spectral imaging captures data at different wavelengths, to characterize various tissue types based on their absorption.
- Multi-phase imaging may be used to obtain images at different points in time, often after application of contrast, to study the uptake and washout of contrast agents, for instance to evaluate synovitis, tumors, or vascular abnormalities.
- Multi-modal imaging relies on various imaging techniques (e.g., MRI, positron emission tomography, computed tomography, and ultrasound) to collect complementary information from these different methods on the same tissue or organ and pathology.

Multifaceted imaging is of particular interest in osteoarthritis research and interventional (DMOAD) trials, because of the heterogeneous nature and etiology of the disease and the involvement of most (peri-) articular tissues. As different MOAs of putative DMOADs may target cartilage, synovium, bone, or other tissues, alone or in combination, multifaceted imaging must be tailored to match this specific MOA by depicting the tissue (components) and pathologies most likely to be modified. Failure to match the image acquisition protocol and “MRI-contrast” to the drug’s MOA will likely result in trial failure, of which there have been plenty (the “graveyard” of DMOAD studies). Of cartilage-modifying drugs, some act anabolic, and some anti-catabolic, with location-independent measurement systems having been developed to determine whether a DMOAD promotes cartilage thickening, prevents thinning, or both [15, 70]. Similar observations can be made for drugs targeting synovitis or bone. However, osteoarthritis participants in clinical trials have often been regarded as homogenous population. Due to inappropriate subtype selection, some trial participants may not respond to the specific DMOAD, reducing the “signal” and enhancing the “noise” in the trial data. This evidently obstructs efficient differentiation of DMOAD vs. placebo-treated patients, causing a failure to demonstrate DMOAD efficacy. Yet, it has to be kept in mind that at the later disease stages, when radiographic alterations are predominant, structural pathologies of the various articular tissues may align and change in a more synchronized manner over time (“final common pathway” or better “common end-stage”) [71], regardless of the initial pathophysiology. At this stage, it may be of less importance which specific tissue is measured, but more important which analytic method displays the greatest precision, reliability, and sensitivity to structural change (of any tissue) within the timelines of the trial. At this stage, the number of measured endpoints may thus be reduced, albeit alignment with the drug’s MOA remains a priority. To comply with regulatory approval of new drugs, “multi-faceted” imaging-derived biomarkers should thus undergo surrogate endpoint qualification [72], allowing them to serve as “short-term” endpoint for longer-term clinical outcomes such (virtual or real) need for surgical joint replacement [73–75].

In conclusion, for a DMOAD trial to be successful, a tight value and precision chain (Fig. 1) must be in place, involving careful patient selection matched to the MOA of the drug studied, a “multifaceted” image acquisition protocol, appropriate (validated and compliant) image analysis tools, and qualified endpoints that can potentially serve as surrogate endpoints, closely related to relevant outcomes. “Multifaceted” MRI does justice to osteoarthritis being a whole-organ disease, involving all joint tissues and a multitude of structural pathologies that offer opportunity for therapeutic intervention. The rigorous application and validation of this value chain will lead to greater effectiveness of clinical trials and drug development by tailoring MOAs, image acquisition protocols, analytic methods, and endpoints to specific “theratypes” [6]. In this context, multifaceted imaging does not only serve as structural endpoint(s), but may also do so for patient selection (precision-medicine). This approach should improve patient diagnosis, prognosis, and clinical management, and lead to more successful DMOADs development programs, effective for most osteoarthritis patients, or certain subgroups (personalized medicine).

## Data Availability

Not applicable.

## Abbreviations

CE: Contrast enhancement
DCE-MRI: Dynamic contrast enhanced magnetic resonance imaging
DESS: Double echo steady state (both acquisitions merged)
DMOAD: Disease-modifying osteoarthritis drug
dGEMRIC: Delayed gadolinium-enhanced magnetic resonance imaging of cartilage
FLAIR: Fluid-attenuated inversion recovery
gagCEST: Glycosaminoglycan chemical exchange saturation transfer
IW: Intermediately weighted
MESE: Multiple echo spin echo
MOA: Mechanism of action
MOAKS: MRI osteoarthritis knee score
MRI: Magnetic resonance imaging
ms: Milliseconds
PD: Proton density
qDESS: Quantitative double echo at steady state (both acquisitions separated)
ROAMES: Rapid osteoarthritis MRI eligibility score
SE: Spin echo
T1: Longitudinal relaxation time
T1rho: T1 relaxation time in the rotating frame
T2: Transverse relaxation time
TE: Echo time
TR: Repetition time
TSE: Turbo spin echo
-w: Weighted

## Declarations

### Disclaimer

Views and opinions expressed are however those of the authors only and do not necessarily reflect those of the European Union or the European Health and Digital Executive Agency (HADEA). Neither the European Union nor the granting authority can be held responsible for them. The content of this work has not been contingent upon approval from the study sponsor. ChatGPT 4.o was used to edit and proof-read parts of the manuscript, and to verify and specify terminology and definitions.
